# A Narrative Review of Weight Management Strategies: From Lifestyle Interventions to Emerging Pharmacotherapies

**DOI:** 10.1155/jobe/6556057

**Published:** 2026-04-13

**Authors:** Iman Saad Ahmed, Sara Luay Tapponi, Hala Malek Manaa, Amina Soltani, Elzahraa Shehata Hussein, Fatemeh Ali Parvaresh, Zahid Hussain

**Affiliations:** ^1^ Department of Pharmaceutics and Pharmaceutical Technology, College of Pharmacy, University of Sharjah, Sharjah, 27272, UAE, sharjah.ac.ae; ^2^ Research Institute for Medical and Health Sciences, University of Sharjah, Sharjah, 27272, UAE, sharjah.ac.ae

**Keywords:** antiobesity drugs, diabetes management, GLP-1 agonists, herbal, intermittent fasting, weight loss

## Abstract

**Background:**

Obesity is a chronic, multifactorial disease associated with significant cardiometabolic, psychological, and socioeconomic consequences. Its complex etiology necessitates integrated, evidence‐based management strategies beyond simple caloric restriction.

**Objective:**

This narrative review aims to critically evaluate current and emerging weight management strategies, including lifestyle interventions, dietary patterns, behavioral approaches, pharmacotherapy, and bariatric procedures, with attention to efficacy, limitations, sustainability, and real‐world applicability.

**Methods:**

A structured literature search was conducted using PubMed, Scopus, ScienceDirect, and Google Scholar to identify relevant peer‐reviewed articles on obesity management. Studies addressing dietary strategies, physical activity, behavioral therapy, antiobesity medications, and surgical or endoscopic interventions were included. Evidence was synthesized narratively, emphasizing comparative effectiveness, safety considerations, and long‐term challenges.

**Results:**

Lifestyle interventions remain the foundation of obesity management; however, long‐term success is often limited by physiological adaptations, behavioral factors, and socioeconomic barriers. Among dietary approaches, differences in weight loss tend to diminish over time, highlighting adherence and sustainability as key determinants of success. Pharmacotherapy has advanced significantly, particularly with incretin‐based agents such as GLP‐1 receptor agonists and dual GIP/GLP‐1 agonists, which produce substantial weight loss and cardiometabolic benefits but require ongoing use and may be limited by cost and tolerability. Bariatric surgery remains the most effective long‐term intervention for severe obesity, while less invasive endoscopic procedures are expanding treatment options. Behavioral and psychological support plays a critical role in improving adherence across all interventions.

**Conclusion:**

Effective obesity management requires a personalized, multidisciplinary approach integrating sustainable lifestyle strategies with pharmacological or procedural therapies when appropriate. While recent therapeutic innovations have expanded treatment possibilities, long‐term success depends on behavioral support, accessibility, and continued evaluation of safety and cost‐effectiveness.

## 1. Introduction

Obesity is one of the most pressing global health challenges increasingly recognized as a leading cause of preventable death worldwide [1, 2]. The World Health Organization (WHO) defines obesity as abnormal or excessive fat accumulation that presents a significant risk to health [3]. This condition arises primarily from prolonged energy imbalance where calorie intake consistently exceeds expenditure, resulting in the accumulation of excess body fat. However, obesity is not merely a matter of caloric surplus; it is a complex, multifactorial disease influenced by genetic, epigenetic, behavioral, and environmental factors. Its management is particularly challenging due to its chronic nature and high recurrence rates, often requiring lifelong interventions. The health implications of obesity are profound, as it significantly increases the risk of severe comorbidities contributing to reduced life expectancy and diminished quality of life [4]. Additionally, it places an immense burden on healthcare systems worldwide, contributing to rising costs and resource strain [5, 6]. Recent estimates suggest that by 2030, obesity and lifestyle‐related diseases could account for up to 30% of global deaths, emphasizing the urgent need for effective prevention and management strategies [3]. Body mass index (BMI) remains the most widely used diagnostic tool for obesity. However, the BMI alone fails to account for variations in muscle mass, fat distribution, and bone density [7]. The aim of this review article is to provide a comprehensive overview of obesity management strategies, examining the efficacy, limitations, and recent advancements in lifestyle interventions, pharmacotherapy, and bariatric surgery, to inform evidence‐based approaches for addressing this global health challenge.

## 2. Methods

### 2.1. Study Search

The literature search was conducted across four databases: ScienceDirect, PubMed, Scopus, and Google Scholar. The main search keywords included “weight loss,” “GLP‐1 agonists,” “herbal supplements,” “intermittent fasting,” “diabetes management,” and “anti‐obesity drugs.” Full‐text articles were retrieved if they were deemed relevant based on title and abstract screening.

### 2.2. Study Selection

**FIGURE 1:**
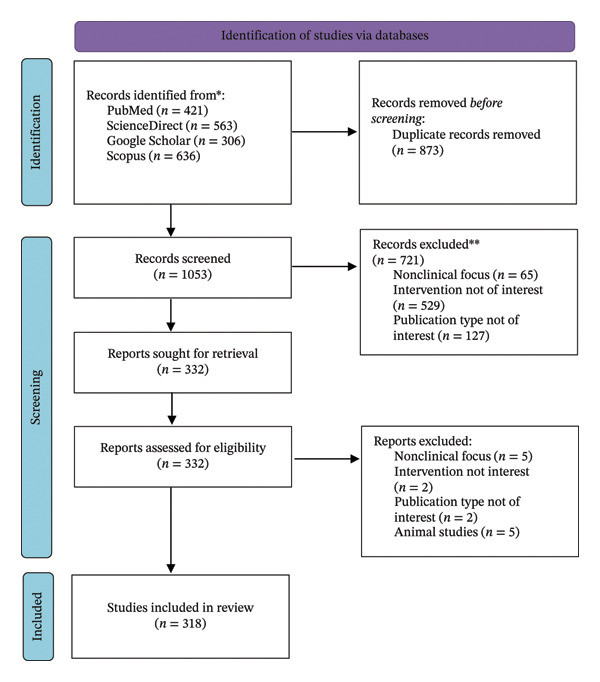
PRISMA‐style flowchart illustrating the literature search and study selection process.

Studies were included if they focused on weight‐loss strategies such as lifestyle modifications, pharmacological interventions (e.g., glucagon‐like peptide‐1 [GLP‐1] receptor agonists [RAs]), dietary approaches, herbal supplements, or surgical procedures like bariatric surgery. Eligible studies encompassed peer‐reviewed original research articles, clinical trials, meta‐analyses, systematic reviews, and high‐quality narrative reviews, published in English with full‐text availability. Studies were excluded if they focused solely on animal models or in vitro experiments without clinical relevance, or if they were conference abstracts, editorials, or commentaries lacking substantial data. Two authors independently screened titles and abstracts for relevance. A total of 318 relevant studies were selected. The literature selection process was documented using a PRISMA‐style flow diagram (Figure 1) to enhance transparency and reproducibility. Records identified through various databases were screened based on the title and abstract, followed by full‐text assessment for eligibility according to predefined inclusion and exclusion criteria.

## 3. Epidemiology

In 2022, 2.5 billion adults aged 18 years and older were classified as overweight, including 890 million who were living with obesity. This indicates that 43% of adults were overweight, and 16% were living with obesity, a sharp increase, as the prevalence of obesity has more than doubled since 1990 [8]. Recent data from the United States (August 2021–August 2023) show that 40.3% of adults aged 20 and older are classified as obese. The prevalence is highest among adults aged 40–59 at 46.4%, followed by 38.9% among those aged 60 and older, and 35.5% in adults aged 20–39 [9]. High BMI accounts for 5 million deaths annually, primarily due to noncommunicable diseases (NCDs) such as diabetes, cardiovascular diseases (CVDs), stroke, and certain cancers. The World Obesity Atlas 2024 highlights alarming projections for the global obesity crisis among both adults and children. By 2035, over 1.77 billion adults are expected to be overweight, and 1.53 billion adults will be living with obesity, representing more than half of the global adult population. This marks a significant rise from 2020, when 42% of adults were affected, a figure projected to increase to 54% by 2035. The crisis also extends to children and adolescents, with more than 750 million children aged 5–19 expected to be overweight or obese by 2035, equivalent to two in every five children globally [1]. In 2022 alone, 390 million children and adolescents were overweight, including 160 million living with obesity. Middle‐income countries are particularly impacted, experiencing higher prevalence rates and associated health consequences [8].

In the Middle East, obesity prevalence remains a pressing issue. A study analyzing data from 2000 to 2020 reported an overall prevalence of overweight at 33.14% and obesity at 21.17% among adults in the region. Syria had the highest obesity rates (40.62%), while Yemen had the lowest (8.8%). The study also highlighted a gender disparity with women exhibiting a higher prevalence of obesity (25.4%) compared to men (19.86%), whereas men were more likely to be overweight [10]. The issue is particularly concerning in the United Arab Emirates (UAE), which ranked 26th globally in obesity prevalence in 2016. A 2019 study found that 17.8% of adults in Dubai are obese, while 39.8% are overweight. Among different population groups, UAE nationals exhibited the highest obesity rates (39.6%), followed by other Arab groups (27.7%). The study also revealed that obesity prevalence increases with age, peaking among individuals aged 50–59 years. Key risk factors for obesity in the UAE included nationality, occupation, and hypertension [11].

### 3.1. Classifications of Obesity

Obesity classification is essential for understanding its health implications and choosing appropriate interventions. Table 1 provides an overview of various methods used to classify obesity, highlighting their key metrics, clinical relevance, and limitations. Obesity is primarily classified using the BMI, a simple calculation based on dividing a person’s weight in kilograms by the square of their height in meters [27]. Despite its popularity, the BMI as a diagnostic tool has certain limitations as it does not account for muscle mass, fat distribution, or body composition [13]. Alternative methods can be used to complement the BMI and overcome its limitations to provide a more accurate obesity classification. For example, waist circumference (WC) and waist‐to‐hip ratio (WHR) provide a clearer picture of central obesity. WC measures the abdominal area around the waist, reflecting fat accumulation in the midsection, while WHR is the ratio of WC to hip circumference, indicating the distribution of fat between the torso and hips [19]. Furthermore, the body adiposity index (BAI) offers a convenient estimation of body fat percentage based on hip circumference and height. However, its accuracy can vary in individuals with extreme body types, such as those with high muscle mass or very high/low body fat percentages, requiring careful interpretation [28]. For a more comprehensive assessment of body composition, advanced imaging techniques provide detailed insights, each with distinct advantages and limitations. Additionally to improve clinical relevance, The Edmonton Obesity Staging System (EOSS) is used to classify obesity based on clinical and metabolic impact rather than body size alone [29].

**TABLE 1:** Obesity classification methods.

Classification method	Description	Key metrics or criteria	Clinical relevance	Limitations	References
Body mass index (BMI)	A ratio of weight (kg) to height (m^2^) used to broadly classify weight status.	< 18.5 (underweight), 18.5–24.9 (normal), 25–29.9 (overweight), ≥ 30, (obesity). Cutoffs differ in some populations.	Commonly used screening tool as it is cheap and effective.	Overestimates risk in muscular individuals; underestimates risk in older adults or those with high visceral fat.	[12–14]

Waist circumference (WC)	Measures abdominal fat by assessing the circumference of the waist.	Abdominal obesity if WC > 102 cm for men and > 88 cm for women.	Indicates central obesity; strongly associated with visceral fat and metabolic risks.	Interindividual variability in measurements; influenced by body shape and posture.	[15–17]

Waist‐to‐hip ratio (WHR)	Compares the circumference of the waist to that of the hips.	High risk: WHR ≥ 0.9 (men) and ≥ 0.85 (women)	Identifies central obesity and correlates with cardiovascular and metabolic disease risks.	Limited accuracy in individuals with extreme body proportions or specific ethnic groups.	[18, 19]

Body adiposity index (BAI)	Estimates body fat percentage based on hip circumference and height.	BAI = (hip circumference/height^1.5^) − 18	Does not require weight measurement; more closely reflects body fat percentage.	Less accurate in individuals with high muscle mass or unique fat distribution patterns. Less accurate than other classification methods.	[20–22]

Body composition imaging techniques	A group of imaging methods, including DEXA, CT, MRI, ultrasound, and PET, used to assess fat mass, fat metabolism, and body composition.	Differentiates fat mass, muscle mass, and subcutaneous vs. visceral fat.	Provides precise body composition analysis and helps identify obesity‐related health risks.	High cost, radiation exposure (for CT and PET), and may have limited accessibility.	[20, 23, 24]

Edmonton Obesity Staging System (EOSS)	Stages obesity based on the severity of comorbidities and functional impairment.	Stage 0: no risk factors; Stages 1–4: progressive severity of comorbidities and disabilities.	Accounts for metabolic, physical, and psychological impacts, enabling personalized obesity management plans.	Requires thorough clinical evaluation, not suitable for quick, or large‐scale population assessments.	[1, 25, 26]

### 3.2. Causes

Compared to other diseases with typically multifactorial and complex etiologies, obesity is more often associated with clearly identifiable causes. It is strongly influenced by individual factors, including diet, physical activity levels, eating patterns, and overall body composition [30]. However, it is important to recognize that a high body weight does not necessarily equate to high fat content, and conversely, a normal weight does not guarantee low fat content [31]. Overweight and obesity result from an imbalance between caloric intake and energy expenditure. When caloric intake consistently exceeds the body’s energy requirements, the excess energy is stored as fat, leading to gradual weight gain and, ultimately, obesity [32]. The primary drivers of the global obesity epidemic are a combination of increased food availability, changes in dietary habits, and a marked decline in physical activity levels [33].

Beyond lifestyle factors, biological mechanisms such as metabolic dysregulation also contribute to obesity. Chronic hyperinsulinemia, often associated with high refined carbohydrate intake, promotes adipose tissue expansion and disrupts energy homeostasis [34]. Hormonal dysregulation also contributes to obesity. Leptin resistance reduces satiety signaling and energy expenditure, while altered ghrelin regulation promotes persistent hunger and difficulty maintaining energy balance [35].

Moreover, the gut microbiota may contribute to obesity through effects on energy harvest, metabolic signaling, bile acid metabolism, and low‐grade inflammation. Experimental studies have demonstrated that microbiota from obese donors can promote increased adiposity when transferred to germ‐free mice, supporting a potential causal role of microbial composition in energy balance [36]. Human studies have also reported associations between obesity and altered gut microbial profiles, and small interventional trials indicate that modifying the microbiota may influence metabolic parameters such as insulin sensitivity [37, 38]. However, findings across populations remain heterogeneous, and no consistent microbiota signature for obesity has been established. Therefore, while the gut microbiome represents a biologically plausible factor in obesity pathophysiology, microbiota‐targeted therapies for weight loss remain investigational and are not yet part of standard clinical management [39, 40]. Several medical conditions also contribute to obesity through different mechanisms, primarily by disrupting hormonal balance and metabolism. For instance, hypothyroidism slows down the metabolic rate, leading to weight gain and reduced energy expenditure [41, 42]. Cushing’s syndrome, marked by elevated cortisol levels, promotes higher insulin levels, enhancing glucose metabolism and fat storage while also increasing appetite and cravings for sugary and salty foods [43]. Similarly, polycystic ovarian syndrome (PCOS) causes weight gain due to insulin resistance and elevated insulin levels [44]. Metabolic syndrome, which includes conditions such as hypertension, insulin resistance, and abdominal obesity, is also associated with increased insulin levels that contribute to weight gain [45, 46].

### 3.3. Risk Factors

#### 3.3.1. Nonmodifiable Risk Factors

Genetics can play a role in determining an individual’s risk of developing obesity. Those include monogenic, polygenic, and syndromic forms [47]. Additionally, family history of metabolic disorders, such as Type 2 diabetes (T2DM) and hypertension, predisposes offspring to early metabolic imbalances, further increasing their risk of developing obesity [48–50]. Parental influence also has a profound impact with a higher maternal BMI being strongly correlated with a greater likelihood of childhood obesity [51, 52]. Furthermore, ethnicity plays a notable role in shaping obesity risk, with studies showing that populations such as Black and Hispanic individuals exhibit a higher predisposition to obesity, likely due to genetic susceptibility coupled with sociocultural influences [53].

#### 3.3.2. Modifiable Risk Factors

Physical inactivity is one of the most significant risk factors for obesity. A sedentary lifestyle, characterized by prolonged periods of inactivity such as excessive screen time, contributes to an energy imbalance, with excess calories being stored as fat [54]. Globally, reduced physical activity is further exacerbated by increasing reliance on transportation and sedentary occupations [55–57]. Excessive caloric intake, particularly from ultraprocessed and energy‐dense foods, represents another major risk factor. These foods, often high in sugar and unhealthy fats but low in nutritional value, drive weight gain and are closely linked to metabolic disorders such as T2DM and CVDs [58, 59].

Environmental factors can also increase obesity risk by shaping behaviors. The abundance of easily accessible, calorie‐dense foods has become a driving force in obesity prevalence. Additionally, certain medications, such as corticosteroids and antipsychotics, can disrupt appetite through similar mechanisms. Careful monitoring of patients at high risk of obesity is essential when prescribing these medications [60, 61].

Furthermore, behavioral and psychological factors play a central role in obesity. Emotional eating, defined as eating in response to negative emotions rather than physiological hunger, is commonly associated with weight gain and difficulty maintaining dietary changes [62]. Stress‐related overeating may be driven by the activation of the hypothalamic–pituitary–adrenal axis, leading to elevated cortisol levels that increase appetite and preference for energy‐dense “comfort” foods [63–66]. Sleep deprivation and mood disorders such as depression and anxiety can further disrupt appetite regulation and dietary adherence [67, 68]. Lifestyle interventions are more effective when combined with behavioral therapy. Approaches such as cognitive behavioral therapy, motivational interviewing, and structured behavioral weight management programs have demonstrated benefit in improving self‐monitoring, coping strategies, and long‐term adherence to dietary and physical activity recommendations [69, 70]. These interventions help individuals identify triggers for overeating, manage stress without food, and develop sustainable habits. Therefore, integrating behavioral and psychological support into obesity management is considered essential for improving long‐term weight maintenance and reducing relapse [71].

### 3.4. Impact of Obesity

#### 3.4.1. Health Impacts

Obesity is strongly linked to a range of serious health complications, significantly increasing the global burden of NCDs. It predisposes individuals to chronic conditions such as T2DM, CVD, nonalcoholic fatty liver disease (NAFLD), gastroesophageal reflux, osteoarthritis, and certain types of cancer, each of which carries substantial morbidity and mortality risks [72–74]. Obesity also promotes hormonal and metabolic changes that can trigger cancers of the breast, colon, and endometrium [75–77]. Beyond these specific conditions, obesity is a major contributor to over 170 million disability‐adjusted life‐years (DALYs) annually, reflecting the profound loss of quality of life it causes [78]. The associated comorbidities not only shorten life expectancy but also impair daily functioning, reducing overall health‐related quality of life. In children, the rise in obesity has led to early onset metabolic and cardiovascular complications, such as nonalcoholic fatty liver disease and elevated blood pressure [79, 80]. These issues frequently persist into adulthood, further increasing the risk for chronic illnesses and exacerbating the lifetime burden of disease.

#### 3.4.2. Economic Impact

Obesity imposes a substantial economic burden through both direct healthcare costs and indirect costs associated with lost productivity, absenteeism, and disability. Direct medical costs arise from increased utilization of healthcare services for obesity‐related conditions, including diabetes, CVD, and musculoskeletal complications, and have been shown to exceed the medical costs of individuals without obesity by a significant margin. In the United States alone, healthcare expenditures attributable to obesity have been estimated at nearly $173 billion annually, excluding the broader societal costs [81].

Beyond healthcare spending, indirect costs such as reduced productivity, absenteeism, presenteeism, and employment disruption constitute an even greater share of the economic burden. Obesity and overweight were estimated to cause approximately $425.5 billion in economic costs to U.S. businesses and employees in 2023, reflecting lost work days and diminished workforce efficiency [82]. Studies across multiple countries consistently find that indirect costs account for a major proportion of total obesity costs, often exceeding direct medical costs highlighting the broader societal impact beyond the health sector [5].

At the macroeconomic level, the global cost of overweight and obesity is projected to reach an estimated $4.32 trillion annually by 2035, equivalent to nearly 3% of global gross domestic product (GDP) if current trends persist [83]. Country‐level analyses similarly show that obesity’s share of national healthcare expenditures varies widely but can represent significant portions of total health spending and labor productivity losses, especially in high income economies [84].

#### 3.4.3. Social and Psychological Impact

Obesity has profound social and psychological consequences, often extending beyond physical health. Societal stigma and discrimination against individuals with obesity deeply affect their mental health, self‐esteem, and professional opportunities, leading to issues such as depression, anxiety, and body image concerns [85]. This stigma can also discourage individuals from seeking timely healthcare, resulting in delayed diagnoses and more advanced disease progression. The impact on children is particularly concerning, as obesity increases their risk of bullying, social exclusion, and negative stereotyping, which can lead to long‐term mental health disorders and poor academic and social development [86]. These challenges often persist into adulthood, perpetuating unhealthy behaviors and weight issues. Cultural norms linking thinness to success and attractiveness further intensify the internalized weight stigma, contributing to disordered eating, social isolation, and diminished quality of life [87].

## 4. Traditional Approaches for Weight Loss

### 4.1. Caloric Deficit

A caloric deficit occurs when the body burns more calories than it consumes, forcing it to utilize stored energy, primarily from fat reserves, to meet its energy demands [88]. Achieving and maintaining a calorie deficit effectively requires an understanding of basal metabolic rate (BMR) and total daily energy expenditure (TDEE). BMR reflects energy required for essential physiological functions at rest, while TDEE accounts for BMR, physical activity, and the energy expended during digestion and metabolism [89–92]. To achieve sustainable weight loss, creating a moderate calorie deficit based on individual TDEE, typically in the range of 400–500 calories per day, is recommended. This approach often results in a steady weight loss of approximately 0.5–1 kg per week, a rate associated with better adherence and long‐term success [93]. However, it is critical to avoid overly aggressive caloric restriction. Consuming fewer calories than the body’s BMR can result in undesirable consequences, including muscle loss, nutritional deficiencies, and reduced metabolic rate, ultimately undermining both health and weight management efforts [94, 95].

A common pitfall of calorie‐deficit diets is focusing solely on caloric intake while neglecting the quality and balance of macronutrients. While weight loss can technically occur with any combination of foods that adhere to the caloric goal, diets dominated by nutrient‐poor, calorie‐dense foods, are unsustainable and detrimental to overall health [96]. On the other hand, emphasizing adequate protein intake supports muscle preservation, enhances satiety, and promotes better adherence to dietary plans [97–99].

### 4.2. Physical Exercise

Regular physical activity is crucial for sustaining weight loss and offers numerous health benefits. It not only contributes to calorie expenditure but also improves metabolic health, body composition, and overall well‐being [100]. The WHO guidelines recommend 150–300 min of moderate‐intensity or 75–150 min of vigorous‐intensity physical activity weekly. They also encourage practical approaches such as incorporating short activity bouts and reducing sedentary behaviors [56]. Physical exercise can be broadly categorized into cardiovascular (aerobic exercise) and weight training (resistance exercise), each offering unique benefits that complement the other. Cardiovascular exercise includes activities such as running, cycling, and swimming, primarily burning calories during the activity itself. It is highly effective for creating a calorie deficit, reducing visceral fat, and improving cardiovascular health [101, 102]. High‐intensity interval training (HIIT), a form of cardio involving short bursts of effort followed by recovery, is particularly efficient in burning calories while preserving lean muscle mass [103, 104]. However, cardio’s postexercise metabolic effects are less pronounced than those of weight training.

Weight training focuses on building and maintaining muscle mass. Although it burns fewer calories during the activity itself, its long‐term effects on metabolism make it essential for maintaining lean muscle during a calorie deficit. This ensures that weight loss primarily comes from fat rather than muscle [105–107]. Resistance training has also been shown to improve insulin sensitivity and reduce risks of chronic conditions such as T2DM [108]. Combining cardio and weight training provides optimal results for weight management. Cardio facilitates immediate calorie burning and fat loss, while weight training enhances metabolic efficiency and muscle preservation [109]. Incorporating two to three weight training sessions weekly alongside regular cardio maximizes fat loss and improves overall fitness and body composition.

Although physical exercise has countless benefits, it is important to note that it accounts for only 10%–30% of TDEE, with the majority stemming from BMR (60%–70%) and the TEF (10%–15%) [110]. A study found that individuals who engaged in either 150 or 300 min of weekly physical activity without adhering to a specific diet achieved less than 2% weight loss of their initial body weight over 6 months [111]. This highlights that while exercise is vital for health, it is insufficient alone for significant weight loss if dietary habits are not addressed.

### 4.3. Diet Plan

Numerous dietary strategies have been studied for weight loss, each differing in macronutrient composition, structure, and behavioral demands. Evidence suggests that long‐term success is strongly influenced by sustained caloric deficit and adherence, in addition to dietary composition. While certain patterns may provide metabolic or satiety‐related advantages, differences in weight‐loss outcomes often narrow over extended follow‐up periods. A common limitation across all dietary strategies is long‐term adherence [112]. Weight regain following initial loss is frequently reported and is influenced by physiological adaptations that promote increased appetite and reduced energy expenditure, as well as behavioral and environmental factors. Diets that are overly restrictive may produce short‐term success but often show higher dropout rates. Therefore, dietary interventions should prioritize flexibility, cultural compatibility, and long‐term sustainability rather than rapid weight loss alone [113].

#### 4.3.1. Whole Plant‐Based Diet (WFPB)

The WFPB diet emphasizes the consumption of vegetables, fruits, legumes, soy products, nuts, seeds, and other plant‐based protein sources, replacing protein typically derived from meat and animal products. In the BROAD study, a randomized control trial (RCT), the WFPB diet produced a greater BMI reduction than controls, with benefits sustained at 6 months [114]. However, adherence to strict plant‐based patterns may be challenging in populations with strong cultural reliance on animal‐based foods. Additionally, poorly planned plant‐based diets may result in deficiencies of vitamin B12, iron, calcium, and omega‐3 fatty acids, requiring careful dietary planning or supplementation [115].

Beyond weight loss, the WFPB diet contributes to broader health benefits, including improvements in blood pressure, lipid profiles, and HbA1c levels. A case study published in 2013 demonstrated significant reductions in these metrics, further validating the diet’s role in improving overall metabolic health [116].

Unlike traditional diets that often rely on a restrictive “eat less” approach, the WFPB diet adopts a more liberating “eat more” philosophy. By prioritizing low‐calorie‐dense, high‐bulk foods, individuals can achieve satiety without the need for meticulous calorie counting. Studies have shown that people following a WFPB diet consume approximately 50% fewer calories compared to those consuming high‐calorie‐dense foods, aiding in weight loss while promoting satiety [117]. Moreover, vegetarian individuals tend to have higher resting energy expenditure when compared to nonvegetarians. This suggests that eating a plant‐based diet can boost metabolism and enhance calorie burning [117, 118].

A common concern about the WFPB diet is its perceived deficiency in protein due to the absence of meat and animal products. However, research indicates that vegetarian diets supply more than adequate protein and amino acids. Incorporating a variety of vegetables, legumes, and grains ensures a balanced intake of essential amino acids, meeting the body’s protein requirements [119]. Additionally, evidence suggests that their benefits are largely mediated through reduced energy density and increased fiber intake rather than unique metabolic effects. Thus, similar outcomes may be achievable with other high‐fiber, minimally processed dietary patterns [120].

#### 4.3.2. Intermittent Fasting (IF)

IF has gained significant attention in recent years as a popular method of calorie restriction. This dietary approach alternates between periods of fasting, lasting a minimum of 12 h, and shorter periods of food consumption.

**FIGURE 2:**
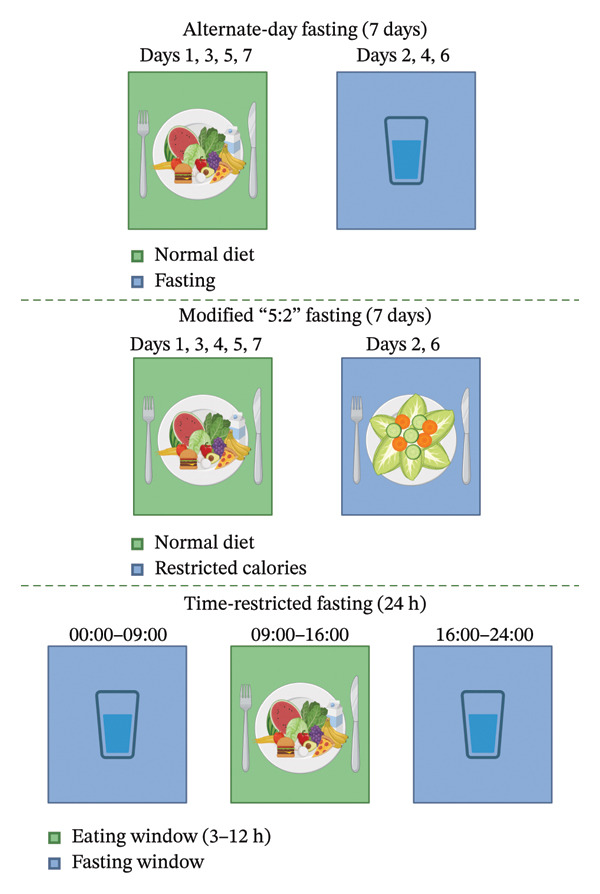
Common intermittent fasting patterns. Created with Biorender.com.

One of the advantages of IF is the flexibility it offers, with several variations to suit individual preferences (see Figure 2). Alternate‐day fasting (ADF) is a method of alternating between a full 24‐h fasting period and a day of normal or restricted calorie consumption. Modified fasting is a subtype of ADF, which involves consuming about 25% of daily caloric requirements on fasting days. This approach, often referred to as the 5:2 method, has been shown to improve blood glucose stability, reduce cravings, and combat fatigue compared to ADF [121–123]. Time‐restricted fasting (TRF) limits food consumption to a specific time window, typically 3–12 h. A meta‐analysis comparing these IF types found no significant difference in their effectiveness for weight loss, suggesting that all three methods can be equally beneficial [124]. Importantly, when total caloric intake is matched, IF does not consistently outperform continuous calorie restriction for weight loss. Its primary advantage may lie in behavioral simplicity rather than unique metabolic effects [125].

The health benefits extend beyond weight reduction. Studies show that IF improves insulin sensitivity, lipid profiles, and markers of inflammation, making it effective in addressing obesity‐related comorbidities [126, 127]. A RCT, INTERFAST‐2, demonstrated a significant reduction in HbA1c levels in participants practicing IF, compared to minimal changes in the control group [128].

Although this diet has demonstrated potential benefits, it is not appropriate for all populations. Vulnerable groups, including older adults, and patients receiving insulin therapy, are generally advised against adopting IF due to the risk of hypoglycemia, inadequate nutrient intake, and potential exacerbation of underlying conditions. Practical implementation may also be challenging, as sustained fasting periods can be difficult to integrate into social routines and daily schedules, potentially affecting adherence [129].

While IF has gained popularity for its simplicity and proposed metabolic effects, the current literature suggests that its weight‐loss effect is comparable to continuous caloric restriction when the total energy intake is matched [125]. However, most studies are short term and conducted under restricted conditions, limiting generalizability to living population. In addition, long‐term adherence remains a challenge, affecting weight‐loss maintenance. From a clinical standpoint, IF may be considered a viable option for patients who prefer a structured eating window, and it should not be promoted as metabolically superior to other types of diets [130].

#### 4.3.3. Low Carbohydrate Diet (LCD)

Carbohydrates are a primary contributor to obesity in many individuals due to their high dietary glycemic load and glycemic index, which stimulate insulin secretion. Insulin, a hormone essential for glucose regulation, also promotes hunger and postprandial glucose fluctuations, further driving weight gain [131, 132]. The LCD addresses this by restricting carbohydrate intake to less than 130 g per day, making it a popular choice for managing obesity and related health conditions such as T1DM, T2DM, and even intractable epilepsy in patients unresponsive to medication [133, 134]. By reducing carbohydrate consumption, LCD helps achieve better glycemic control and promotes the secretion of satiety hormones like GLP‐1 and PYY, which aid in appetite regulation [131, 135, 136].

Furthermore, the LCD serves as the foundation for several widely recognized dietary approaches, including the zone diet, Atkins diet, South Beach Diet, and the ketogenic diet (KD) [135]. The most popular one is the KD, which is a very low carbohydrate diet (VLCD) that involves reducing the carbohydrates intake to less than 50 g per day while relatively increasing the proportions of protein and fat [137]. Originally introduced to manage epilepsy, KD induces a metabolic state called nutritional ketosis, in which the body produces ketone bodies from fat to replace glucose as the primary energy source [138, 139]. In a RCT, patients on KD reduced their HbA1c by 0.9% from baseline, compared to a reduction of 0.5% in the control group, while achieving an average weight loss of 8.5 kg over 16 weeks compared to a loss of 3.9 kg in the control group [140]. Additionally, KD has shown efficacy in managing conditions such as T2DM, PCOS, CVDs, and neurological disorders, with meta‐analyses confirming significant reductions in BMI, fasting glucose, and HbA1c [141].

Despite its benefits, KD is not without risks and limitations. Evidence suggests that the initial rapid weight loss seen with LCD is partly due to glycogen depletion and associated water loss [142]. Over longer follow‐up periods, weight‐loss differences between low carbohydrate and other calorie‐restricted diets tend to diminish. This can also be due to poor adherence in free living conditions due to the restrictive nature of LCD. High dropout rates in long‐term studies raise serious concerns about sustainability outside controlled settings [143]. Patients on insulin or other antidiabetic medications may face an increased risk of hypoglycemia and diabetic ketoacidosis, requiring careful medication adjustments before initiating the diet. Moreover, patients on sodium‐glucose cotransporter 2 (SGLT2) inhibitors should avoid KD entirely due to the heightened risk of diabetic ketoacidosis [144, 145]. The diet may also worsen lipid profiles in certain individuals and poses risks of nephrolithiasis and metabolic acidosis, making it unsuitable for patients with chronic kidney disease, cardiac arrhythmia, or elderly patients with frailty [122, 139, 146]. Common short‐term side effects include gastrointestinal (GI) discomfort, fatigue, polyuria, and “keto breath,” which may affect adherence despite often being transient [135, 139, 147].

#### 4.3.4. Mediterranean Diet (MedDiet)

The MedDiet is characterized by a high intake of plant‐based foods, legumes, and olive oil, moderate consumption of fish and poultry, and limited intake of red meat and refined carbohydrates. Evidence supports modest but clinically meaningful weight reduction, particularly when implemented as an energy‐restricted pattern and paired with physical activity. In a meta‐analysis of RCTs, MedDiet interventions were associated with greater reductions in body weight and BMI compared with control diets, with stronger effects observed when the MedDiet was calorie restricted and maintained for ≥ 6 months [148].

Longer duration RCTs further support its effectiveness and practicality. The two‐year DIRECT trial (322 participants) found that a MedDiet produced weight loss comparable to low carbohydrate approaches [149]. More recently, the PREDIMED‐Plus trial, which is a large lifestyle intervention using an energy restricted MedDiet plus physical activity, has reported a significantly greater weight loss at 12 months compared with a control MedDiet advice group (mean difference ≈ −2.5 kg) [150].

Moreover, MedDiet benefits extend beyond weight. In PREDIMED (primary prevention), MedDiets supplemented with extra virgin olive oil or nuts reduced major cardiovascular events [151]. However, weight‐loss effects are typically moderate, and outcomes depend heavily on adherence, baseline diet quality, and whether calorie restriction is explicitly implemented. Some heterogeneity also reflects a variation in how MedDiet is operationalized across studies, which complicates direct comparison between trials [152].

#### 4.3.5. Dietary Approaches to Stop Hypertension (DASH) Diet

The DASH diet emphasizes fruits, vegetables, whole grains, lean proteins, and low‐fat dairy while limiting saturated fat, added sugars, and sodium [153]. Although not designed primarily as a weight loss diet, RCTs and meta‐analyses have demonstrated that adherence to the DASH pattern can support modest reductions in body weight and BMI, when coupled with caloric restriction and lifestyle modification. A meta‐analysis of RCTs reported greater weight loss and BMI reduction in adults following the DASH diet compared with controls over 8–24 weeks [154] Clinical evidence also indicates that the DASH diet’s impact on weight is often intertwined with broader lifestyle changes. In the ENCORE trial, the DASH diet combined with aerobic exercise and behavioral weight management produced significant weight loss (∼19 lb) and improvements in metabolic biomarkers, whereas the DASH diet alone without caloric restriction yielded only minimal weight loss and modest metabolic changes [155]. Beyond weight, the DASH diet exerts well‐established effects on cardiometabolic risk factors. Systematic reviews and meta‐analyses demonstrate significant reductions in systolic and diastolic blood pressure, total and LDL cholesterol, and other cardiovascular risk markers [154, 156]. However, the certainty of evidence for some outcomes (e.g., glycemic control and long‐term weight maintenance) remains moderate to low, and the magnitude of weight changes is generally modest compared with more restrictive or structured weight‐loss diets. Variability in trial outcomes is present from differences in sodium restriction, baseline cardiometabolic status, and the presence or absence of concurrent energy restriction. As such, the DASH diet may be particularly valuable in individuals with obesity who also have hypertension or elevated cardiovascular risk, serving as a clinically targeted dietary strategy rather than a universal weight‐loss solution [157].

### 4.4. Herbal Intervention

Herbal‐ and plant‐derived compounds continue to attract scientific and public interest as potential adjuncts in weight management due to their biological plausibility and accessibility. Experimental and clinical studies suggest that certain botanicals may influence appetite regulation, fat metabolism, or energy expenditure [158]. However, the overall strength of evidence remains inconsistent. Many clinical investigations involve relatively small sample sizes, short intervention periods, heterogeneous formulations, and variable dosing strategies, which limit comparability and generalizability. In addition, long‐term efficacy and safety data are often insufficient, and publication bias toward positive findings cannot be excluded. As a result, herbal supplements are best considered supportive rather than primary strategies within comprehensive obesity management [159].

#### 4.4.1. Green Tea

Green tea contains catechins, particularly epigallocatechin gallate (EGCG) and caffeine, which are thought to modestly increase energy expenditure and fat oxidation [160, 161]. Its effectiveness for weight loss has been evaluated in several studies with mixed results. In certain populations, such as women with PCOS, green tea has shown promise. A systematic review of four RCTs found that green tea supplementation significantly reduced body weight (mean difference: −2.80 kg) [162]. However, a Cochrane Systematic Review examining the effects of green tea in obese adults found no significant weight loss compared to placebo [163]. Additionally, a systematic review and meta‐analysis of green tea supplementation combined with exercise found small but consistent reductions in weight and BMI compared to exercise alone, suggesting that while green tea may enhance the effects of exercise, its overall impact on weight loss remains limited [164].

Although generally safe at moderate intake, high‐dose green tea extracts, particularly those rich in EGCG, have been associated with hepatotoxicity. Doses linked to weight loss in some studies often exceed typical dietary intake, raising safety concerns. Many trials are short in duration and vary in formulation and dosing, limiting conclusions about long‐term efficacy and safety. As such, green tea may provide modest adjunctive benefit, but its clinical impact on weight loss is small [165, 166].

#### 4.4.2. Cinnamon Supplementation

Cinnamon, a well‐known spice derived from the inner bark of trees in the Cinnamomum genus, contains active compounds such as cinnamaldehyde, eugenol, and cinnamic acid. The primary active compound, cinnamaldehyde, activates the transient receptor potential ankyrin 1 (TRPA1) receptor, which is involved in regulating energy expenditure and fat oxidation. Through this mechanism, cinnamaldehyde increases thermogenesis, delays gastric emptying, and reduces ghrelin, all of which contribute to reduced food intake and improved fat metabolism [167]. Research suggests that cinnamon supplementation may have promising effects on weight management, body composition, and metabolic health. A systematic review and meta‐analysis of 12 RCTs involving 786 participants found that cinnamon intake significantly reduced body weight, BMI, WC, and fat mass [168]. Complementing these findings, an umbrella meta‐analysis of seven studies demonstrated weight and BMI reduction with cinnamon, particularly at doses of 3 g/day or higher [169]. However, considerable heterogeneity in cinnamon species, preparation methods, and dosing across studies limits comparability and prevents firm clinical recommendations. Cinnamon may provide modest adjunctive benefits, but current evidence is insufficient to support its use as a primary weight loss intervention.

#### 4.4.3. Garcinia Cambogia (GC)

GC, also known as Garcinia gummi‐gutta, is a tropical fruit native to Southeast Asia and parts of Africa. It contains hydroxycitric acid (HCA), which has been proposed to affect appetite and lipid metabolism [170]. A systematic review and dose response meta‐analysis reported small reductions versus placebo in body weight (−1.34 kg) and BMI (−0.99 kg/m^2^), though heterogeneity across trials limits clinical certainty [171]. More recent randomized trials in women with NAFLD have evaluated HCA as an adjunct to a calorie‐restricted diet; one trial reported reductions in visceral adiposity without significant changes in leptin/adiponectin [172], and another trial found improvements in anthropometric measures and metabolic/atherogenic biomarkers when HCA was combined with calorie restriction [173]. Concerns about the safety of GC, particularly hepatotoxicity, have been raised. Cases of liver injury linked to the use of GC, either alone or in combination with other supplements, have been documented [174–176]. The presence of the HLA‐B∗35:01 allele has been suggested as a genetic predisposition for this liver toxicity, indicating that individual susceptibility may play a role [177]. Given the generally modest and variable weight loss effects observed in clinical studies, together with reported risks, GC should be approached cautiously and is not considered a primary weight management strategy.

#### 4.4.4. Others

Several other plant‐derived compounds have been studied as adjuncts to lifestyle‐based weight management. Capsaicin and capsaicinoids (from chili peppers) have been evaluated in RCTs; a systematic review and meta‐analysis suggest modest reductions in body weight, BMI, and WC in overweight or obese adults, with effect sizes generally small and heterogeneity across trials [178–182]. Ginger has also been investigated; recent meta‐analyses of RCTs report small improvements in body weight and body composition parameters, although findings vary by dose, duration, and population. [183, 184]. Curcumin and turmeric extracts have shown small but statistically significant reductions in the BMI, body weight, and WC in pooled analyses; however, clinical relevance for weight loss alone is typically modest [185–187]. Among botanicals with more “metabolic” positioning, berberine has emerging higher quality clinical evidence. A recent multicenter RCT in individuals with obesity and metabolic dysfunction–associated steatotic liver disease (MASLD) assessed the effects on visceral adiposity and liver fat over 6 months, supporting potential benefits on adiposity‐related outcomes beyond short‐term surrogate markers [188–190]. Finally, psyllium husk is often used as a soluble fiber supplement to support satiety and cardiometabolic risk reduction. Meta‐analytic evidence suggests psyllium’s direct effect on body weight is small or inconsistent, but it has more consistent evidence for improving lipid and cardiometabolic risk markers [191, 192]. Collectively, these supplements may offer incremental benefits in selected patients, but they should be framed as adjuncts rather than primary weight loss interventions, with attention to formulation variability, tolerability, and patient‐specific cardiometabolic goals.

## 5. Pharmacotherapy

Despite their efficacy, lifestyle modifications sometimes fail to achieve significant and sustainable weight loss in individuals with obesity due to several challenges. These include low adherence rates, physiological adaptations, and compensatory hormonal changes. As a result, there has been a growing demand for effective pharmacotherapy to support sustainable weight management. According to the AACE/ACE obesity guidelines, pharmacotherapy is considered a second‐line treatment, recommended when lifestyle modifications alone prove insufficient to achieve or sustain clinically significant weight loss [193]. Table 2 provides a comprehensive overview of pharmacological therapies for obesity, highlighting their key characteristics, clinical profiles, and therapeutic benefits.

**TABLE 2:** Overview of pharmacotherapy for obesity management.

Drug name	Mechanism of action	Dosage	Route of administration	Safety profile	FDA approval date for obesity	Key clinical trials	Unique advantages	Efficacy (% average weight loss) over 1 year	References
Orlistat	Pancreatic and gastric lipase inhibitor; reduces dietary fat absorption	120 mg three times daily with meals	Oral	GI SE (oily stools, flatulence), fat‐soluble vitamin deficiencies	1999	XENDOS	Nonsystemic action; available OTC in lower dose	∼10% over 1 year	[194–197]
Phentermine/topiramate	Phentermine: sympathomimetic amine; topiramate: anticonvulsant with appetite suppression	Initial: 3.75 mg/23 mg once daily; maintenance: 7.5 mg/46 mg once daily	Oral	Insomnia, dry mouth, constipation, paresthesia; teratogenic risk; potential for increased heart rate	2012	CONQUER, SEQUEL	Higher efficacy compared to monotherapy; extended‐release formulation for once‐daily dosing	∼10% over 1 year	[198–201]
Naltrexone/bupropion	Naltrexone: opioid receptor antagonist; bupropion: norepinephrine‐dopamine reuptake inhibitor	8 mg/90 mg tablet; two tablets twice daily after titration	Oral	GI SE, increased blood pressure; boxed warning for suicidal thoughts and behaviors	2014	COR	Addresses both appetite and reward pathways; beneficial for patients with depressive symptoms	5%–10% over 1 year	[202–204]
Liraglutide	GLP‐1 receptor agonist; increases satiety and slows gastric emptying	Start with 0.6 mg once daily, increase weekly by 0.6 mg to reach 3.0 mg	Subcutaneous injection	GI SE, pancreatitis risk; boxed warning for thyroid C‐cell tumors in rodents	2014	SCALE obesity and prediabetes	Improved glycemic control; cardiovascular benefits observed in trials	∼8% over 1 year	[205–208]
Semaglutide	GLP‐1 receptor agonist; increases satiety and slows gastric emptying	2.4 mg once weekly after titration	Subcutaneous injection	GI SE, pancreatitis risk; boxed warning for thyroid C‐cell tumors in rodents	2021	STEP	Higher efficacy compared to other GLP‐1 RAs; once‐weekly dosing	> 15% over 1 year	[209–211]
Tirzepatide	Dual GLP‐1 and GIP receptor agonist; enhances insulin secretion, reduces appetite	Start at 2.5 mg weekly, increase to 10–15 mg weekly	Subcutaneous injection	GI SE; potential for hypoglycemia when used with insulin or insulin secretagogues	2023	SURMOUNT	Superior efficacy; dual action; once‐weekly dosing	∼20% over 1 year	[212–214]
Cagrilintide/semaglutide	Cagrilintide: amylin analog; Semaglutide: GLP‐1 receptor agonist; both increase satiety	—	Subcutaneous injection	Mild hypoglycemia; Mild or moderate GI SE	—	Ongoing clinical trials	Potential for enhanced efficacy through dual mechanism; once‐weekly dosing	15%–17.1% over 20 weeks	[215, 216]
Mazdutide	Dual GLP‐1 and glucagon receptor agonist; increases energy expenditure and reduces appetite	—	Subcutaneous injection	GI SE; potential for increased heart rate; further safety profile under investigation	—	Glory 1; Ongoing	Targets both appetite suppression and energy expenditure; potential for higher efficacy	—	[217]
Retatrutide	Triple agonist (GLP‐1, GIP, and glucagon receptors); enhances insulin secretion, reduces appetite, increases energy expenditure	—	Subcutaneous injection	GI SE; potential for increased heart rate; further safety profile under investigation	—	Ongoing clinical trials	Multireceptor targeting for potentially superior efficacy; once‐weekly dosing	24.2% over 48 weeks	[218]
Orforglipron or danuglipron	Small‐molecule GLP‐1 receptor agonist; enhances insulin secretion, suppresses glucagon, and slows gastric emptying	—	Oral	GI SE; further safety profile under investigation	—	Ongoing clinical trials	Oral administration without injection or fasting restrictions	Orforglipron: 9.4%–14.7% over 36 weeks. Danuglipron: 8%–13% over 32 weeks	[219, 220]

Although pharmacological therapy has evolved substantially in recent years, antiobesity medications (AOMs) should be interpreted within the context of important clinical and practical limitations. Most agents require long‐term or lifelong use to maintain weight reduction, as discontinuation frequently leads to partial or complete weight regain. Additionally, tolerability, contraindications, cost, and variability in individual response often limit real‐world effectiveness compared to outcomes observed in RCTs [221].

### 5.1. Orlistat

Orlistat is a reversible lipase inhibitor that was first approved in 1999 for prescription use under the brand name Xenical for obesity management in conjunction with a reduced caloric diet and to reduce the risk of regaining weight after prior weight loss. Subsequently, in 2007, the FDA approved a lower‐dose, over‐the‐counter version of orlistat, marketed as Alli, for weight loss in overweight adults aged 18 and older, to be used in conjunction with a reduced‐calorie, low‐fat diet [197]. It is recommended for patients with a BMI greater than 30 kg/m^2^ or a BMI greater than 28 kg/m^2^ with obesity‐related comorbidities such as hypertension, diabetes, hyperlipidemia, and obstructive sleep apnea. Due to its minimal systemic absorption and noncentral nervous system activity, orlistat is particularly safe for long‐term use, including in patients with CVDs or antipsychotic‐induced weight gain [222–224].

Orlistat has been extensively studied in both adults and adolescents. In a RCT in adolescents, orlistat reduced BMI by 0.55 and WC, while the placebo group experienced a BMI increase of 0.31 [225, 226]. In adults, a 4‐year trial (XENDOS) demonstrated significant weight reduction (mean 5.8 kg with orlistat vs. 3.0 kg with placebo) and a lower incidence of diabetes (6.2% vs. 9.0% in the placebo group), confirming its benefits in reducing diabetes risk and maintaining glycemic control [194]. However, the magnitude of weight loss with orlistat is modest compared with newer agents, and GI adverse effects frequently lead to poor adherence in clinical practice.

The recommended doses of Alli and Xenical are 60 mg and 120 mg taken three times daily with each main meal containing fat or within 1 hour of eating. Discontinuation or restarting does not require dosage adjustments [227, 228]. To enhance GI tolerability, experts may start patients on the 60 mg orlistat dose or switch to it if the 120 mg dose causes side effects [229]. Patients should supplement with multivitamins, as orlistat can reduce the absorption of fat‐soluble vitamins (A, D, E, K) by inhibiting pancreatic lipase, which is essential for hydrolyzing vitamin esters [230].

Orlistat’s adverse effects are primarily GI and include oily stools, flatulence, abdominal discomfort, and fecal incontinence, which are associated with its pharmacological mechanism and may impact adherence [231]. To enhance GI tolerability, experts may start patients on the 60 mg orlistat dose or switch to it if the 120 mg dose causes side effects [229]. Furthermore, while orlistat is often described as cost‐effective, its long‐term effectiveness is highly dependent on sustained dietary modification, which remains challenging for many patients outside structured programs [230].

### 5.2. Phentermine/Topiramate (PT)

PT was approved in 2012 as the first combination medication for long‐term weight management [232]. Phentermine is a sympathomimetic amine working as an appetite suppressant, although the exact mechanism is not fully understood. While effective for short‐term weight loss, phentermine monotherapy was limited due to its potential for abuse and cardiovascular side effects, including tachycardia, hypertension, and pulmonary hypertension. As a result, it is contraindicated in patients with a history of CVDs [198]. Topiramate, a gamma‐aminobutyric acid (GABA) receptor agonist traditionally used as an anticonvulsant, has been shown to induce significant weight reduction in epileptic patients [225].

When these two drugs are combined, they offer a substantial reduction in weight. The CONQUER trial, a randomized controlled study, evaluated the efficacy of PT in obese adults with related comorbidities. Over 56 weeks, patients receiving 15/92 mg and 7.5/46 mg doses achieved mean weight reductions of 10.2 kg and 8.1 kg, respectively, compared to a weight reduction of 1.4 kg in the placebo group [200]. Building on the findings from the CONQUER trial, the SEQUEL study demonstrated that the combined use of controlled‐release PT with lifestyle modifications achieved substantial and sustained weight loss over 108 weeks. Patients receiving doses of 15/92 mg and 7.5/46 mg experienced mean weight reductions of 10.5% and 9.3% from baseline, respectively, compared to a reduction of 1.8% in the placebo group. PT has also proven effective in treating obesity in adolescents over the age of 12 years [233]. In a double‐blinded randomized trial, adolescents receiving 15/92 mg experienced a 10.4% reduction in the BMI percentage, while those on 7.5/46 mg and placebo showed reductions of 8.4% and 0.2%, respectively [234].

Common adverse effects include dry mouth, constipation, sinusitis, headaches, insomnia, and paresthesia and psychiatric side effects such as depression, anxiety, and impaired concentration [225, 235]. PT is administered orally, once daily, typically in the morning to reduce the risk of insomnia [200]. The medication is contraindicated in individuals with glaucoma, hyperthyroidism, hypersensitivity to sympathomimetic amines, or those who have taken monoamine oxidase inhibitors (MAOIs) within 14 days of discontinuation [236]. Concerns remain regarding its sympathomimetic component, particularly in patients with underlying cardiovascular risk. Careful patient selection and monitoring are essential, and its use may be limited in older adults or those with established heart disease. These safety considerations, along with regulatory restrictions in some countries, limit the widespread use of this therapy despite its strong efficacy profile.

### 5.3. Naltrexone/Bupropion (NB)

NB, marketed under the brand name Contrave, is an FDA‐approved medication for long‐term obesity management. It combines two active ingredients, naltrexone, a μ‐opioid‐receptor antagonist (RA), and bupropion, an antidepressant with dopamine and norepinephrine reuptake inhibition effects [237]. Naltrexone enhances the anorexigenic effects of bupropion, creating a synergistic effect that aids in obesity management [225, 230, 238, 239].

The COR‐behavioral modification (BMOD) trial evaluated the efficacy of sustained‐release NB as an adjunct to BMOD. Patients on NB + BMOD achieved a weight loss percentage greater than 9.3 ± 0.4% compared to 5.1 ± 0.6% in the placebo + BMOD group, demonstrating the efficacy of NB for obesity management therapy as an adjunct to BMOD [240]. In another study, it was observed that obese patients treated with NB had a significant reduction in Hb1Ac (0.6%) when compared to those treated with placebo (0.1%). This suggests that NB can be effective in managing obesity‐related diabetes [241].

NB outperforms orlistat in terms of effectiveness but is less effective than PT. However, it offers the advantage of a lower potential for abuse compared to PT [242]. The most common adverse effect is nausea, but other side effects include constipation, headaches, insomnia, dizziness, and dry mouth. To minimize the occurrence of nausea, it is recommended to start with a small dose and increase it gradually. It is important to note that while bupropion has antidepressant properties, NB is not a treatment for mood disorders, and psychiatric adverse effects, including anxiety, insomnia, and suicidal ideation, warrant careful monitoring, particularly in vulnerable individuals [243]. Moreover, it is contraindicated in patients with chronic opioid use, acute opioid withdrawal, abrupt cessation of alcohol or sedatives (e.g., benzodiazepines), uncontrolled hypertension, seizure disorders, or recent use of MAOIs [236]. Additionally, the modest weight loss achieved with NB compared with incretin‐based therapies has led to reduced clinical preference in recent years, especially when balanced against its side‐effect burden and contraindications.

### 5.4. GPL‐1 Receptor Agonist: A Novel Antiobesity Pharmacotherapy

**FIGURE 3:**
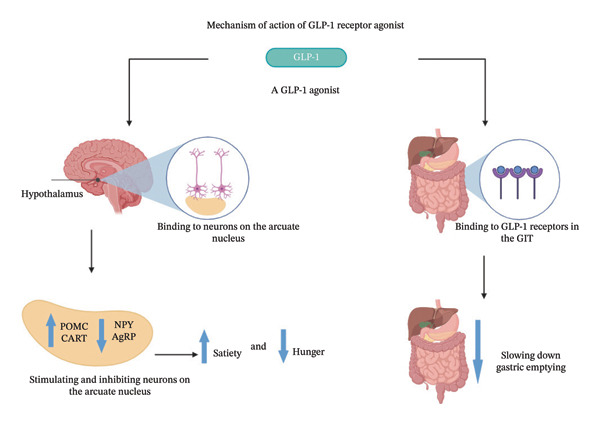
Mechanism of action of GLP‐1 receptor agonists. Created with Biorender.com. Abbreviations: AgRP: Agouti‐related peptides ; CART, cocaine and amphetamine regulated transcript; GLP‐1, glucagon‐like‐peptide 1; NPY, neuropeptide Y; POMC, proopiomelanocortin.

Pharmacotherapy has long been recognized as a viable option for weight management. Recent advancements, however, have significantly improved its efficacy, making it more appealing to both healthcare providers and patients. Clinical trials have demonstrated that newer drugs, like GLP‐1 RAs, can achieve substantial and sustained weight loss (Figure 3) [244–246]. Recent FDA approvals of these medications, originally indicated for diabetes but now repurposed for weight management, have enhanced their credibility and fast acceptance. Furthermore, social media platforms have amplified awareness and interest, with personal success stories shared by users and influencers accelerating their popularity [247]. Despite their impressive efficacy, GLP‐1 receptor agonists are associated with important limitations. GI intolerance remains the most common reason for discontinuation, particularly during dose escalation [248]. In addition, rapid weight loss may include reductions in lean body mass, emphasizing the importance of concurrent nutritional and resistance‐training strategies [249]. Long‐term safety data extending beyond several years are still emerging, especially for newer, higher‐dose formulations.

#### 5.4.1. Liraglutide

Liraglutide, one of the newer GLP‐1 RAs, offers a significant advantage over earlier drugs like exenatide, which has a much shorter half‐life of approximately 2.4 h, by exhibiting an extended half‐life and enhanced efficacy [250].

Liraglutide was initially approved by the FDA in 2010 for the management of T2DM under the brand name Victoza. Building on its effectiveness in improving glycemic control and promoting weight loss, liraglutide was later approved in 2014 under the brand name Saxenda for chronic weight management in adults. It became the first GLP‐1 RA approved specifically for obesity, marking a significant milestone in pharmacotherapy for this condition. In 2020, this indication was expanded to include pediatric patients aged 12 years and older with obesity [251, 252]. Liraglutide acts on the arcuate nucleus of the hypothalamus, a critical brain region for appetite regulation, to suppress hunger‐promoting (orexigenic) pathways and activate satiety‐promoting (anorexigenic) pathways to reduce caloric intake [253]. Additionally, liraglutide slows gastric emptying, prolonging the feeling of fullness after meals, further aiding in appetite control. The SCALE Obesity and Prediabetes study, a Phase 3 clinical trial involving 3731 participants with a BMI ≥ 30 or ≥ 27 with comorbidities, evaluated liraglutide (3.0 mg) versus placebo alongside lifestyle interventions over 56 weeks. Participants receiving liraglutide achieved a placebo‐adjusted mean weight loss of 5.6 kg, with 63.2% attaining at least 5% body weight reduction, compared to 27.1% in the placebo group [206]. Similarly, a Canadian study reported a mean weight reduction of 6.5%–7.1% over 6 months, with over 64% of the participants achieving at least a 5% reduction and 34.5% losing over 10% of their body weight [254]. These consistent findings highlight liraglutide’s effectiveness in both clinical trials and real‐world settings for weight management. Furthermore, liraglutide has shown promising results in pediatric populations, as highlighted in a recent review on its efficacy in treating obesity in children and adolescents [255]. For instance, the SCALE Teens trial demonstrated a 4.64% BMI reduction in adolescents over 56 weeks, showing its potential as a therapeutic option for severe obesity in younger populations when combined with lifestyle interventions [256].

The most common side effects of liraglutide are GI symptoms, such as nausea, vomiting, and diarrhea, especially at higher doses. These can lead to dehydration and, in rare cases, acute kidney injury. Elevated serum lipase and amylase levels raise concerns about acute pancreatitis, though the absolute risk remains low. Gallbladder‐related issues, including cholelithiasis, have been reported due to reduced gallbladder contractility. Mild increases in the heart rate are also noted, likely from direct receptor stimulation. Rare hepatic effects, such as elevated liver enzymes and autoimmune hepatitis, have been observed [257, 258]. Close monitoring and appropriate dosing are critical to balance efficacy and safety. Furthermore, daily subcutaneous administration represents a significant barrier to long‐term adherence, and real‐world persistence rates are substantially lower than those reported in clinical trials [259].

#### 5.4.2. Semaglutide

Semaglutide is another GLP‐1 RA developed to address the adherence challenges posed by liraglutide’s daily injections. Initially approved by the FDA in 2017 under the brand name Ozempic for T2DM, its indication was expanded to include weight management in 2021 with the approval of Wegovy [260, 261].

Comprehensive evidence of semaglutide’s efficacy in weight management has been shown through the STEP trial program, a series of Phase 3 RCTs designed to evaluate its effects on obesity. Across these trials, semaglutide consistently outperformed placebo and demonstrated clinically meaningful weight loss in individuals with and without T2DM. For example, in STEP 1, participants with obesity but no diabetes achieved a mean weight loss of 14.9% from baseline after 68 weeks of treatment with semaglutide 2.4 mg, compared to 2.4% with placebo. Moreover, 86.4% of participants receiving semaglutide achieved at least 5% weight loss, with significant reductions also observed across higher thresholds including 15% and 20% weight loss [262]. In STEP 2, a trial involving individuals with both T2DM and obesity, semaglutide 2.4 mg yielded an average weight loss of 9.6%, outperforming both the 1.0 mg dose (7.0%) and placebo (3.4%) [263]. STEP 4 highlighted the necessity of continued therapy, as the discontinuation of semaglutide after 20 weeks resulted in significant weight regain [264]. Meanwhile, the longer‐term STEP 5 trial demonstrated that weight loss with semaglutide 2.4 mg was sustained over 2 years, with participants achieving a mean reduction of 15.2% from baseline [265]. Despite this, distinct patterns emerge in terms of adverse events. A systematic review found that while both drugs commonly caused mild to moderate GI side effects, such as nausea and diarrhea, semaglutide had a greater frequency of total adverse events but fewer severe outcomes. Liraglutide demonstrated a higher incidence of serious adverse events compared to semaglutide, including severe GI disturbances requiring medical intervention or rare cardiovascular complications. Semaglutide also posed a slightly higher risk of hypoglycemia, particularly in patients on glucose‐lowering therapies, but its overall safety profile was more favorable, with serious complications occurring less frequently than with liraglutide [266].

Ongoing research aims to expand the applications of semaglutide in obesity management. The oral formulation of semaglutide, marketed under the brand name Rybelsus, and initially approved for T2DM, has been evaluated for weight loss efficacy through the OASIS clinical trial program. The OASIS 1 trial demonstrated that once‐daily oral semaglutide 50 mg achieved a mean weight loss of 15.1% over 68 weeks, compared to 2.4% in the placebo group. Additionally, 85% of participants receiving oral semaglutide achieved a weight loss of 5% or more, versus 26% in the placebo group [267]. However, higher doses required for oral efficacy may increase GI side effects, and long‐term comparative data versus injectable formulations are still limited.

#### 5.4.3. Danuglipron and Orforglipron

Danuglipron and orforglipron are novel, orally administered, small‐molecule GLP‐1 RA for the treatment of T2DM and obesity [219, 268]. By eliminating the need for injections, these drugs aim to provide the therapeutic benefits of GLP‐1 RA while improving patient adherence and accessibility.

As of January 2025, neither drug has received FDA approval for weight loss. Danuglipron has shown promising results in Phase 2b trials, with mean placebo‐adjusted weight reductions ranging from 8% to 13% at 32 weeks. However, high discontinuation rates exceeding 50% were observed across all doses, primarily due to GI side effects, highlighting how tolerability may limit real‐world effectiveness despite promising weight loss efficacy in controlled settings [269]. Consequently, Pfizer is focusing on developing a once‐daily modified release formulation to improve the tolerability profile [270]. Eli Lilly’s orforglipron is currently in Phase 3 clinical trials. Phase 2 studies reported body weight reductions ranging from 8.6% to 12.6% at 26 weeks and 9.4%–14.7% at 36 weeks [219]. Phase 3 results are anticipated by mid‐to‐late 2027, with potential FDA approval applications to follow, depending on trial outcomes.

The safety profiles of both drugs suggest no increased risk of serious adverse events or hypoglycemic events when compared to other GLP‐1 RAs, which is encouraging for their use in clinical practice. However, GI‐related side effects remain a significant challenge, as they are the primary reason for treatment discontinuation [270–272]. Despite this issue, the development of these oral formulations holds promise for expanding access to effective obesity treatments. Long‐term studies are needed to thoroughly assess their efficacy, safety, and tolerability. before widespread clinical adoption.

### 5.5. Dual and Triple Hormone Receptor Agonists: A New Frontier in Pharmacotherapy

Emerging advancements in obesity pharmacotherapy have introduced unimolecular dual and triple hormone receptor agonists, which represent a revolutionary weight loss management. Unlike conventional mono‐agonist therapies, these novel drugs simultaneously activate multiple metabolic pathways and demonstrate superior weight reduction and improve glycemic control [273].

#### 5.5.1. Dual Hormone Receptor Agonists

Tirzepatide, the first FDA‐approved dual GLP‐1 and glucose‐dependent insulinotropic polypeptide (GIP)_RA as a treatment for T2DM, has shown a superior weight loss effect and reduction in HbA1c when compared to GLP‐1 RAs alone. Its weight loss effect is due to enhanced insulin secretion, suppressed appetite, and enhanced energy expenditure [274]. Tirzepatide, often referred to as a “twincretin,” is the first and only dual agonist that targets the receptors of two incretin hormones [275]. This novel approach represents a significant advancement in obesity pharmacotherapy [274]. Tirzepatide was first approved by the FDA in May 2022 under the brand name Mounjaro for T2DM treatment, and in October 2023, it received approval as Zepbound for chronic weight management [276, 277]. This approval is for adults with obesity or overweight who have at least one weight‐related comorbidity, to be used in combination with a calorie‐restricted diet and increased physical activity. It is administered as a once‐weekly subcutaneous injection, with a half‐life of approximately 5 days, making it convenient for long‐term use [276].

The efficacy of tirzepatide in weight management has been extensively validated through the SURMOUNT clinical trial program. In SURMOUNT‐1, a Phase 3 RCT involving 2539 adults with obesity or overweight and no diabetes, tirzepatide demonstrated dose‐dependent weight reductions of 15.0% with 5 mg, 19.5% with 10 mg, and 20.9% with 15 mg, compared to a reduction of 3.1% in the placebo group over 72 weeks. In this study, 57% of the participants receiving the highest dose achieved a reduction of 20% or more in baseline body weight, far surpassing results seen with the placebo group [212]. Moreover, a comparative analysis between tirzepatide and semaglutide, using data from the SURMOUNT‐1 and STEP 1 trials, showed that tirzepatide achieved superior weight loss outcomes and a greater likelihood of achieving weight reduction thresholds [278].

As with other incretin‐based therapies, tirzepatide’s most common adverse effects were GI symptoms. These symptoms increase with higher doses, affecting up to 49% of patients at the 15 mg dose. Severe adverse events, including hypoglycemia and pancreatitis, are rare, occurring in less than 1% of cases [279]. Rare cases of thyroid C‐cell tumors have been reported in animal studies, but no such cases have been observed in humans to date [280]. The long‐term safety profile of dual incretin agonism is still under evaluation, and cost considerations may limit accessibility in many healthcare systems.

#### 5.5.2. Triple Hormone Receptor Agonists

Building on dual hormone receptor agonists, triple receptor agonists, targeting GLP‐1, GIP, and glucagon receptors, are currently in clinical development, offering a more comprehensive approach to metabolic regulation. By combining the anorectic and insulinotropic effects of GLP‐1 and GIP and with induced energy expenditure effect due to glucagon secretion, these agents promote fat metabolism while maintaining glucose homeostasis. Early clinical trials suggest that triple agonists may achieve a superior weight reduction and metabolic improvements than current treatments, making them a potential next‐generation solution for obesity management. Ongoing clinical trials aim to establish their safety, efficacy, and long‐term impact on obesity and metabolic disorders [281, 282].

Retatrutide (LY3437943) is an investigational triple hormone receptor agonist currently undergoing clinical trials for obesity. Its mechanism action is through the activation of GLP‐1, GIP, and glucagon receptors simultaneously [283]. In addition to weight reduction mechanisms observed in dual agonists, glucagon receptors activation further decreases food intake, slows down GI time, and enhances energy expenditure [284]. Clinical trials from Phase 1 to 3 have demonstrated the significant efficacy of retatrutide in weight reduction, improving glycemic control and decreasing cardiovascular risk in susceptible patients [283].

In a randomized, double‐blind Phase 2 clinical trial, all retatrutide treatment groups demonstrated a significant reduction in bodyweight from baseline after 36 weeks, with a least‐squares mean reduction of up to 16.94%, while the placebo group had only 3% reduction. This highlights retatrutide strong efficacy in reducing bodyweight [285].

The safety profile of retatrutide is closely related with that of GLP‐1 and GLP‐1/GIP dual agonist, with GI‐related adverse effect being commonly reported [286]. In a Phase 2 clinical trial, the most common GI adverse effects included nausea, diarrhea, vomiting, and constipation, particularly during the dose escalation phase [218]. As these agents remain in early‐ to mid‐stage clinical development, long‐term safety, cardiovascular outcomes, and real‐world tolerability are not yet established. Therefore, their role in clinical practice remains speculative pending further evidence.

### 5.6. Challenges Associated With AOMs

AOMs face several challenges, including uncertainties about their long‐term safety and efficacy. For many individuals, these medications may not sustain weight loss over time, as discontinuation often results in weight regain [287]. Another concern is their misuse for cosmetic purposes by those who do not meet the eligibility criteria, diverting them from their intended use in managing obesity‐related health conditions. Economic barriers, including high costs and limited insurance coverage, further complicate access for eligible patients [288]. Newer AOMs are often associated with high monthly expenses, which may exacerbate health disparities by disproportionately limiting use among individuals of higher socioeconomic status. Cost‐effectiveness analyses remain limited and may vary significantly across healthcare systems. Additionally, the variability in individual responses to AOMs highlights the need for personalized treatment approaches [288]. In summary, while AOMs can be an important tool in obesity management, they are not a cure‐all, and their use should be part of a comprehensive strategy addressing the root causes and complications of obesity.

## 6. Bariatric Surgery

The 2022 guidelines from the American Society for Metabolic and Bariatric Surgery (ASMBS) and the International Federation for the Surgery of Obesity and Metabolic Disorders (IFSO) recommend MBS for individuals with a BMI over 35 kg/m^2^, regardless of comorbidities. Additionally, MBS is advised for patients with metabolic diseases and a BMI between 30 and 34.9 kg/m^2^ [289]. It is particularly advised for patients who have failed to achieve significant and sustained weight loss through lifestyle modifications, behavioral interventions, or pharmacological treatments.

**FIGURE 4:**
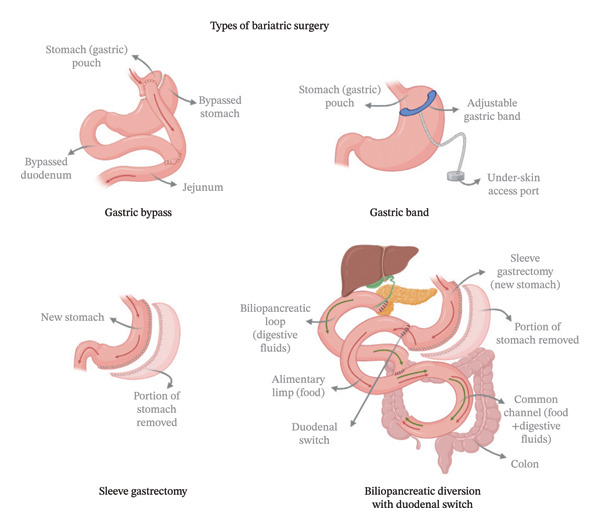
Types of bariatric surgeries. Created with Biorender.com.

Exclusion criteria for bariatric surgery include the presence of endocrine disorders, psychiatric conditions, poor myocardial reserve, severe respiratory dysfunction or COPD, severe gastroesophageal reflux disease (GERD) unresponsive to treatment with proton pump inhibitors (PPIs), symptomatic hiatal hernia, history of metabolic or abdominal surgery, and any history of alcoholism or drug addiction [290, 291]. Table 3 and Figure 4 compare the most popular and widely performed types of bariatric surgeries.

**TABLE 3:** Comparison of different types of bariatric surgeries.

Type of bariatric surgery	Description	Risks	Length of hospital days	Average % of excess weight loss after 1 year	References
Gastric bypass	Resection of the stomach to create a small pouch. From the pouch there is a nonabsorptive bypass outlet connecting it to the intestine.	• Deficiencies in Iron, Vit B12, Vit D, calcium, and folate leading to osteoporosis, anemia, and neuropathy. • Gallstones • Dumping syndrome • Gastric ulcers • Intestinal obstruction	Average of 2.5 days	50%–70%	[290, 292–297]

Sleeve gastrectomy	Vertical resection of the great curve of stomach to reduce 80% of the stomach volume, limiting the intake of food	• GERD • Barret’s esophagus • Wernicke’s encephalopathy • Gastric stenosis • Hemorrhage	2–4 days	56%	[291, 294, 298–300]

Gastric band	An inert band will be placed around the proximal part of the stomach to restrict it without causing malabsorption.	• Tubing malfunction or band erosion and slippage • Gastric perforation • Necrosis of stomach • Aspiration pneumonia • Bolus obstruction	Average of 1.3 days	30%–52%	[290, 294, 295, 297, 301, 302]

Biliopancreatic diversion with duodenal switch	The process involves longitudinal removal of the great curvature of the stomach (aids in caloric restriction) and a duodenal dissection. gastrojejunostomy and jejunojejunostomy are also performed to create a long common channel (reduction of fat and protein absorption) and alimentary limb (reduces caloric absorption)	• Deficiencies in iron, Vit B12, Vit D, calcium, and folate leading to osteoporosis, anemia, and neuropathy • Gastrointestinal leakage • Duodenal stomal obstruction • Gallstones formation	Average of 3.3 days	61.6%–87%	[292, 303–308]

The ultimate aim of these bariatric surgeries is weight loss through either restriction or malabsorption techniques [290]. However, there are other health benefits associated with bariatric surgery. A study suggested that gastric bypass and biliopancreatic diversion are superior to conventional medications in controlling blood glucose levels in obese T2DM patients. While medical therapy resulted in a reduction of 14.37% in glucose levels from baseline, biliopancreatic diversion and gastric bypass achieved reductions of 56.23% and 37.81%, respectively [309]. Bariatric surgery is also associated with reduced incidence of CVDs in obese patients by improving cardiovascular health factors [310]. Malabsorptive surgeries, in particular, have a significant impact on lipid profiles. Studies have demonstrated reductions in total cholesterol, LDL, and triglycerides following such procedures. A meta‐analysis reported a mean LDL reduction of 22.0 mg/dL in surgical patients compared to 4.3 mg/dL in nonsurgical controls [311, 312]. Additionally, bariatric surgery provides mechanical improvements, like reducing weight bearing stress on joints and improving sleep apnea due to decreased fat around the neck as well as improved quality of life and psychosocial status [290].

Although bariatric surgery is the most effective weight loss strategy, it is not universally applicable and most of the supporting evidence is derived from long‐term observational cohorts rather than RCTs with conventional pharmacotherapies. Surgical risks, nutritional deficiencies and long‐term medical follow‐up, should be carefully weighed against benefits [313]. In clinical practice, bariatric surgery should be reserved for medically selected patients, who failed to achieve weight loss through other means.

## 7. Strengths and Limitations

This review provides a comprehensive overview of obesity management strategies, encompassing lifestyle and dietary interventions, behavioral approaches, pharmacotherapy, and bariatric procedures. A major strength lies in its clinically oriented approach, which contextualizes emerging therapies, particularly incretin‐based pharmacotherapies within the framework of long‐term obesity management rather than isolated weight loss outcomes. By comparing safety, efficacy, sustainability, and applicability across the various intervention approaches, this review aims to support informed and patient‐tailored clinical decision‐making.

Nevertheless, several limitations of this review should be acknowledged. As a narrative review, this work is inherently subject to selection and reporting bias and does not include a formal risk‐of‐bias assessment. The summarized evidence for each section is heterogenous, containing RCTs, observational studies, reviews with variable follow‐up durations, defined outcomes and population characteristics limiting direct comparisons across intervention categories. Furthermore, some studies evaluating dietary strategies, herbal supplements, and newer AOMs are relatively of short duration, affecting conclusions about long‐term safety, durability of weight loss, and effectiveness in real‐life clinical settings. These limitations highlight the need for careful interpretation of findings and the significance of future high‐quality and long‐term comparative and cost‐effective studies to guide in the selection of the most appropriate and individualized weight loss strategy.

## 8. Conclusion

Obesity remains a complex, chronic disease with significant health and economic consequences, requiring comprehensive and sustained management strategies. While lifestyle interventions, including dietary modification, physical activity, and behavioral support, remain the foundation of treatment, long‐term success is often limited by physiological adaptation, environmental influences, and variability in individual response. Emerging pharmacological therapies have expanded treatment options and can produce clinically meaningful weight loss, but their benefits must be balanced against cost, tolerability, long‐term safety considerations, and the need for continued use to maintain results. Surgical and endoscopic procedures remain the most effective interventions for severe obesity but require careful patient selection and long‐term follow‐up. Overall, effective obesity management requires an individualized, multidisciplinary approach that integrates sustainable lifestyle strategies with pharmacological or procedural therapies when appropriate. Future efforts should focus on improving long‐term adherence, expanding access to effective treatments, and generating strong long‐term data to guide clinical decision‐making.

## Author Contributions

Conceptualization, Iman Saad Ahmed, Amina Soltani, Elzahraa Shehata Hussein, and Fatemeh Ali Parvaresh; methodology, Iman Saad Ahmed, Amina Soltani, Elzahraa Shehata Hussein, Fatemeh Ali Parvaresh, and Zahid Hussain; validation, Iman Saad Ahmed, Sara Luay Tapponi, and Hala Malek Manaa; formal analysis, Amina Soltani, Elzahraa Shehata Hussein, and Fatemeh Ali Parvaresh; investigation, Iman Saad Ahmed, Sara Luay Tapponi, and Hala Malek Manaa; resources, Iman Saad Ahmed and Zahid Hussain; data curation, Sara Luay Tapponi, Hala Malek Manaa, and Zahid Hussain; writing–original draft preparation, Iman Saad Ahmed, Amina Soltani, Elzahraa Shehata Hussein, Fatemeh Ali Parvaresh, and Zahid Hussain; writing–review and editing, Sara Luay Tapponi, and Hala Malek Manaa; visualization, Iman Saad Ahmed, Sara Luay Tapponi, and Hala Malek Manaa; supervision, Iman Saad Ahmed and Zahid Hussain; project administration, Iman Saad Ahmed.

## Funding

No funding was received for this research.

## Disclosure

All authors have read and agreed to the published version of the manuscript.

## Consent

The authors have nothing to report.

## Conflicts of Interest

The authors declare no conflicts of interest.

## Data Availability

Data sharing is not applicable.
